# Accurate Micromanipulation of Optically Induced Dielectrophoresis Based on a Data-Driven Kinematic Model

**DOI:** 10.3390/mi13070985

**Published:** 2022-06-23

**Authors:** Gongxin Li, Zhanqiao Ding, Mindong Wang, Zhonggai Zhao, Shuangxi Xie, Fei Liu

**Affiliations:** 1 Key Laboratory of Advanced Process Control for Light Industry (Ministry of Education), Institute of Automation, Jiangnan University, Wuxi 214122, China; 6191913013@stu.jiangnan.edu.cn (Z.D.); 6211924106@stu.jiangnan.edu.cn (M.W.); gaizihao@jiangnan.edu.cn (Z.Z.); fliu@jiangnan.edu.cn (F.L.); 2 State Key Laboratory of Robotics, Shenyang Institute of Automation, Chinese Academy of Sciences, Shenyang 110016, China; 3 School of Electrical and Mechanical Engineering, Pingdingshan University, Pingdingshan 467000, China; shuangxi881228@126.com

**Keywords:** optically induced dielectrophoresis (ODEP), data-driven modeling, controller design, accurate manipulation

## Abstract

The precise control method plays a crucial role in improving the accuracy and efficiency of the micromanipulation of optically induced dielectrophoresis (ODEP). However, the unmeasurable nature of the ODEP force is a great challenge for the precise automatic manipulation of ODEP. Here, we propose a data-driven kinematic model to build an automatic control system for the precise manipulation of ODEP. The kinematic model is established by collecting the input displacement of the optical pattern and the output displacements of the manipulated object. Then, the control system based on the model was designed, and its feasibility and control precise were validated by numerical simulations and actual experiments on microsphere manipulation. In addition, the applications of ODEP manipulation in two typical scenarios further demonstrated the feasibility of the designed control system. This work proposes a new method to realize the precise manipulation of ODEP technology by establishing a kinematic model and a control system for micromanipulation, and it also provides a general approach for the improvement of the manipulation accuracy of other optoelectronic tweezers.

## 1. Introduction

Optically induced dielectrophoresis (ODEP), as a micromanipulation technology, has been widely used for micro/nanoparticles’ separation, manufacture and assembly [1,2,3,4]. It is also considered as a label-free and non-invasive technique for the manipulation of living cells. For example, Yang et al. completed the dynamic manipulation of a single microparticle and demonstrated the human liver cells’ patterning for the first time using ODEP [5]; Liao et al. realized the high-purity CD45^neg^/Ep-CAM^neg^ cells’ isolation from the blood samples of cancer patients by ODEP [6]; Liang et al. used ODEP to rapidly separate Burkitt’s lymphoma cells and red blood cells without markers [7]. Our previous works also used the ODEP to isolate *S. cerevisiae* from its surroundings for the diagnosis of several special diseases [8,9,10]. The principle of ODEP manipulation is based on the dielectrophoretic force generated by dynamically altering virtual electrodes that produce non-uniform electric fields applied on polarized particles via controlling the optically projected patterns, which are digitally generated by a computer. The dielectrophoretic force is also called the “ODEP force”, and the ODEP force applied on a spherical particle in a fluidic medium is described as:

(1) FODEP=2πR3εmRe[K*(ω)]∇|E|2

where *R* is the particle radius, *ε_m_* denotes the permittivity of the liquid medium, K*(*ω*); is a Clausius–Mossotti (CM) factor and *E* is the electric field [11,12,13,14,15]. However, the unmeasurable characteristic of ∇*E* in Equation (1) results in it being hard to calculate the ODEP force applied on the manipulated object, which brings great challenges to its precise manipulation. Stoke’s law is often used to characterize the magnitude of the ODEP force, that is, the viscous resistance of the manipulated object when it moves at a uniform speed is equal to the ODEP force [16]. While the manipulated object is also subjected to other forces in addition to the ODEP force, such as gravity, Brown force and viscous resistance [17,18], which makes precise manipulation of ODEP more difficult.

Fortunately, an automated manipulation method provides a feasible solution to the aforementioned technical problem. Our previous work demonstrated an automatic method for ODEP manipulation by introducing image processing and recognition to realize automatic operation on microparticles [19]; Gan et al. also introduced PI control theory into the ODEP system and accomplished specific trajectory control for a single polystyrene microsphere as well as rapid aggregation operations through a visual feedback control method [20]. These automatic operations take the operated position as the controlled object, and use the position deviation of the manipulated particle as the feedback signal to achieve precise operation by adjusting the position of the input optical pattern. In this paper, to acquire more precise automatic manipulation of ODEP, we propose a data-driven kinematic model of ODEP manipulation, which is independent of traditional ODEP force models (Equation (1)) and is established with an ODEP automation control system to achieve more accurate manipulation. A target recognition and location information collection system was designed at first to obtain the displacements of the manipulated particles and the optical patterns based on the previous works [19]. Then, the collected data were used to establish the kinematic model of ODEP manipulation. The controller and automatic control system were designed to achieve precise manipulation, and the latter’s feasibility and control precision were validated by the numerical simulation. Finally, several experiments have verified that the system achieved high-precision operations in different applications. This provides a new method to describe the kinetic mechanisms of ODEP manipulation for precise operations, and it is also used as a general approach for the improvement of the manipulation accuracy of other optoelectronic tweezers.

## 2. Materials and System

### 2.1. Sample Preparation

5 μm polystyrene microsphere solution is prepared as follows. 7 mL deionized water and 3 μL microsphere solution (Shanghai Huge Biotechnology Inc., Shanghai, China) with the concentration of ~9 × 10^6^ beads/mL were dropped in a test tube, and then, the solution undergoes ultrasonic treatment for 30 min. In addition, add 400 μL bovine serum albumin (BSA) solution to the microsphere solution to reduce its friction with ITO conductive glass and its adhesion to the base plate of the ODEP chip [7,19]. The prepared microsphere solution undergoes ultrasonic treatment for 10 min before experiments.

### 2.2. Experimental Setup

**Figure 1:**
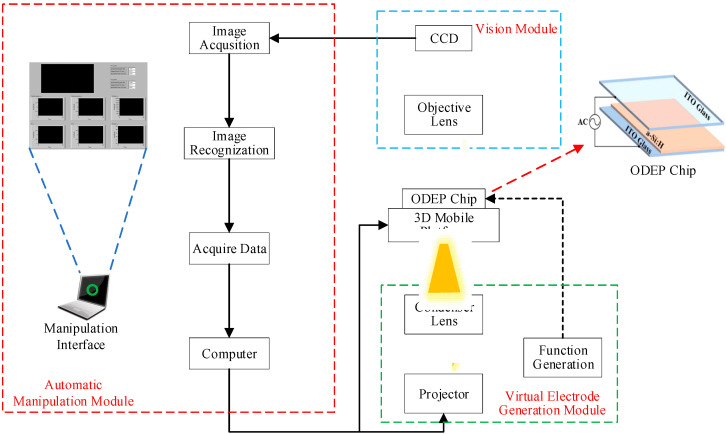
Schematic of the experimental system.

The system mainly includes a vision module, ODEP chip, 3D mobile platform, virtual electrode generation module and automatic manipulation module, as shown in Figure 1. The vision module is composed of a charge coupled device (CCD) (PL-D755, PixeLink Inc., Ottawa, ON, Canada) and an objective lens, and is used to observe the manipulation process in real time. The ODEP chip is the core part of the ODEP system, in which the micromanipulation is performed and it is fixed on a 3D mobile platform. The virtual electrode generation module consists of a projector, a condenser lens and a function generator. A projector is used to project optical patterns drawn by the software onto the ODEP chip, and then a function generator is used to apply AC voltage to both the upper and lower layers of the indium tin oxide (ITO) glasses of the ODEP chip to generate a virtual electrode in the solution layer, consequently generating a non-uniform electric field and ODEP force applied on the manipulate particles. The automatic manipulation module contains the functions of data collection, target recognition and the automatic setting of optical patterns [19]. During automatic operation, the automatic manipulation module identifies the manipulated object at first by an image recognition method, and then automatically captures and moves the object to the target location by the designed control system.

### 2.3. Fabrication of ODEP Chip

As the core part of the ODEP system, the ODEP chip is a “sandwich” structure, as shown in Figure 1, whose upper and lower layers are transparent conductive glass covered with ITO on one side (impedance of 3 Ω). The lower layer of the ITO glass is deposited with a photosensitive material of hydrogenated amorphous silicon (a-Si:H) (thickness of 500 nm) on the side attached with ITO by the plasma enhanced chemical vapor deposition method (PECVD) [17,21]. Then, the photosensitive material on the surface of the lower layer was scratched off the at the boundary at 2~3 mm wide to expose the ITO conductive layer, which is used to connect with the AC voltage. The two layers of the ITO glasses are cleaned with ethanol and blow dried with nitrogen, respectively, and they are bonded together with double-sided scotch tape to assemble a complete ODEP chip. Then, take two copper wires: one end of the two wires is fixed on the conductive part of the two-layer ITO glass substrate with adhesive copper foil, and the other end is clamped with the function generator, respectively. Finally, the chip is fixed on the 3D mobile platform.

### 2.4. Working Principle of ODEP System

The a-Si:H is a well photoelectric material that has a large impedance under the unilluminated condition, and shares most of the AC voltage drop, while the voltage of the middle layer of an ODEP chip is low, that is, not enough to generate a non-uniform electric field to actuate manipulated objects. When an optical pattern designed by the drawing software is projected on the surface of the a-Si:H layer of ODEP chips, the impedance of the illuminated area as well as its surrounding decreases significantly, which generates a non-uniform electric field in the middle layer [22,23]. Then, an ODEP force will be produced and applied on manipulated objects in the middle layer, so as to realize the manipulation of the objects.

In this work, annular optical patterns are chosen to capture and manipulate 5 μm polystyrene microspheres. The relative position of the manipulated microsphere in the optical pattern is affected by the direction of the ODEP force, which is determined by the CM factor. Namely, if the microsphere is subjected to a positive ODEP force, it moves to the edge of the illuminated area, and then, attaches to the edges of the annular optical pattern; on the contrary, the moving direction of the microsphere is towards the center of the illuminated area, and thus, a potential well is created under the action of negative ODEP forces that keeps the microsphere in the center of the optical pattern [24,25]. So, in order to manipulate the microsphere more easily, a negative ODEP force is chosen and adjusted in the following results by changing the medium conductivity, where the microsphere is captured in the center of the annular optical pattern.

## 3. Data-Driven Modeling

### 3.1. Data Acquisition

Establishing a kinematic model has a vital impact on improving the control accuracy of the ODEPs’ manipulation. Although the system has a mechanism model for the ODEP force as described above, the immeasurability of ∇*E* in the model makes it more difficult to achieve precise control of the manipulation [26]. Therefore, a kinematics model of the ODEP manipulation with a controllable input and measurable output is needed to achieve precise operational control. To establish the model, the microsphere in the view is arbitrarily selected to be manipulated, and the corresponding operation parameters are set, including the optical pattern and its moving speed; then, the manipulated process in real-time is recorded through the vision module, and the displacement data of the optical pattern and the manipulated microsphere are extracted and collected. Finally, these data are divided into two parts, one of which is used to build a data-driven kinematic model, while the other is used to verify the accuracy of the model.

Usually, an annular optical pattern is used to capture and manipulate a single microsphere, and the optical pattern is controlled to move and push the manipulated microsphere to the target location. Here, the displacements of the optical pattern are used as input signals, and the displacements of the manipulated microsphere are taken as output signals.

**Figure 2:**
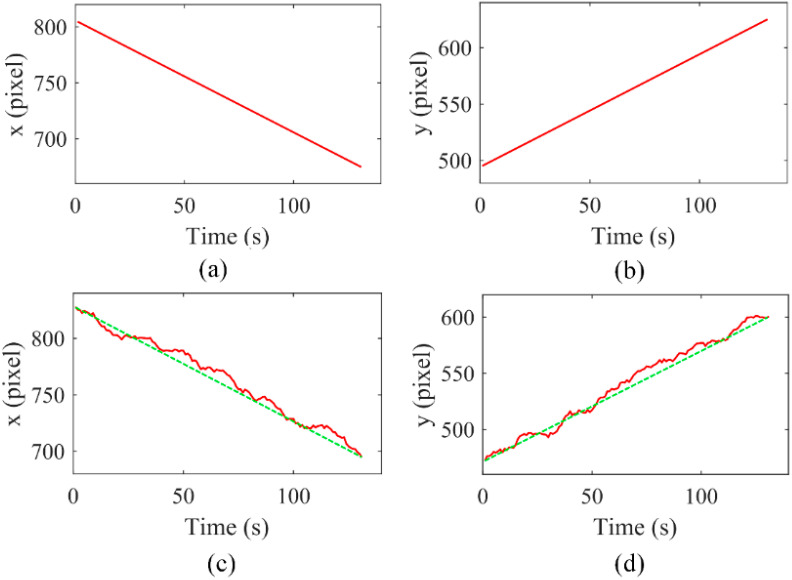
An example of data acquisition. Coordinates values of the optical pattern at the x-axis (**a**) and the y-axis (**b**), respectively. Corresponding coordinates values of the manipulated microsphere at the x-axis (**c**) and the y-axis (**d**), respectively. The green dashed lines represent the ideal movement trajectories of the manipulated particle.

Combining with our previous work on automatic manipulation [19], the input and output datasets were obtained by using the imaging recognition method to automatically identify and record the relative positions of the manipulated microsphere at the different moving speeds of the optical pattern. The inner radius and the outer radius of the annular optical pattern are 20 μm and 25 μm, respectively, and the movement speeds of the optical patterns vary from 0.75 μm/s to 2.0 μm/s. All acquired data are under the same magnification of the CCD. The position information of the optical patterns and manipulated microspheres is based on the pixel value of the field of view as the position coordinates. The coordinates are established with the lower left corner of the field of view as the origin, the horizontal direction as the x-axis and the vertical direction as the y-axis. Figure 2 is a typical dataset of the collected signals, which includes the time-domain input data of the x-axis and y-axis values of the optical pattern (Figure 2a,b) and the corresponding output data of the manipulated microsphere (Figure 2c,d). Figure 2c,d also show that there are uncertain deviations in the movement trajectory of the manipulated microsphere between the real-time measured state and in the ideal state (only subject to the ODEP force), mainly because the microsphere is affected by forces other than the ODEP force, including viscous resistance, gravity, etc.

### 3.2. Modeling

The kinematic model of the ODEP system takes the displacements of the optical patterns as the input and the locations of the manipulated objects as the output, and the model is described as the following by Equation (2).

(2) $$\left[\begin{array}{l} y_{1}(k)\\ y_{2}(k) \end{array}\right]=G(z^{-1})\left[\begin{array}{l} u_{1}(k)\\ u_{2}(k) \end{array}\right]$$

where *u_i_*(*k*), *i* = 1,2, are the coordinate values of the x-axis and y-axis of the optical pattern, respectively; *y_i_*(*k*), *i* = 1,2 and indicate the coordinate values of the x-axis and y-axis of the manipulated microsphere, respectively; and *G*(*z*^−1^) represents the transfer function.

Then, the collected dataset is divided into two parts, of which 600 sets of the data are used to build the model and the remaining 219 sets of the data are used to verify the accuracy of the model. The system identification toolbox of Matlab software was used to established the transfer function *G*(*z*^−1^), as described in Equation (3).

(3) $$G(z^{-1})=[\begin{array}{c} \frac{1.038-1.02z^{-1}}{1-0.9797z^{-1}-0.003785z^{-2}}\\ \frac{0.0007-0.0007089z^{-1}}{1-1.998z^{-1}+0.9988z^{-2}} \end{array}\begin{array}{c} \frac{-0.0002807+0.0002092z^{-1}}{1-1.993z^{-1}+0.9938z^{-2}}\\ \frac{1.008-1.008z^{-1}}{1-1.04z^{-1}+0.04001z^{-2}} \end{array}]$$

Considering the independent control of the x-axis and y-axis, Equation (3) can be simplified as Equation (4):

(4) $$G(z^{-1})=[\begin{array}{c} \frac{1.038-1.02z^{-1}}{1-0.9797z^{-1}-0.003785z^{-2}}\\ 0 \end{array}\begin{array}{c} 0\\ \frac{1.008-1.008z^{-1}}{1-1.04z^{-1}+0.04001z^{-2}} \end{array}]$$

**Figure 3:**
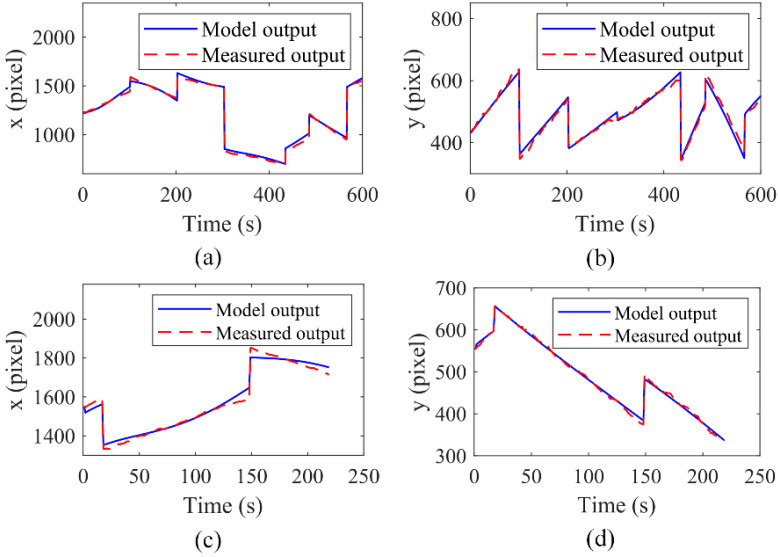
The model output and the measured output with the datasets for modeling at the x-axis (**a**) and the y-axis (**b**), respectively; The model output and the measured output with the data sets for validating the model at the x-axis (**c**) and the y-axis (**d**), respectively.

Figure 3a,b show the measured data and the model output data of the x-axis and y-axis in the process of establishing the model, respectively, and the red dotted lines and the blue solid lines in Figure 3 represent the actual measured output and model-calculated output of the manipulated microsphere in the same input trajectory of the optical pattern, respectively. The best fit at the x-axis and y-axis are 93.36% and 84.18%, respectively. Figure 3c,d show the corresponding model verification curves, which were acquired by validating the model with the remaining 219 datasets at the x-axis and y-axis, respectively. The results show that the model accuracy at the x-axis and y-axis are 86.37% and 94.46%, respectively. The results demonstrate that the established model can fit well the relationship between the input and output of the system.

### 3.3. Controller Design

**Figure 4:**
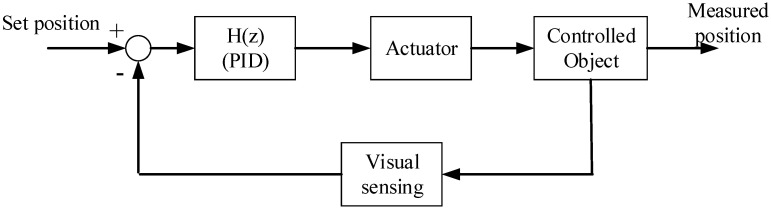
Schematic diagram of the control system.

Based on the aforementioned kinematic model, an automated operation controller was established. PID, as a precise control method commonly used in industrial fields and embedded equipment, was adopted in this control system, as shown in Figure 4. Considering that the data used for modeling are time-domain data in two vertical directions, and the data in the two directions themselves are decoupled, therefore, an independent PID control process for the two directions were established, and the transfer function is demonstrated in Equation (5):

(5) $$H(z)=\left[\begin{array}{cc} PID_{1}(z) & 0\\ 0 & PID_{2}(z) \end{array}\right]$$

In addition, it must be considered that the manipulated microsphere should always be located inside the optical pattern during the operation process when using the annular optical pattern, otherwise, there will be an escape phenomenon, that is, if the manipulated microsphere is outside the annular during the movement, it is difficult to capture it again and realize target operation. Especially, escape of the manipulated microsphere is easier to occur when the feedback deviation value is larger than the diameter of the annular optical pattern. Therefore, the PID output is not directly used as the input value of the controlled object, and instead, the initial values are added, as described in Equation (6), that is,

(6) $$\left[\begin{array}{c} u_{1}\\ u_{2} \end{array}\right]=\left[\begin{array}{c} v_{1}\\ v_{2} \end{array}\right]+\left[\begin{array}{c} x_{0}\\ y_{0} \end{array}\right]$$

where *v*_1_ and *v*_2_ are the PID output of the x-axis and y-axis, respectively, and (*x*_0_,*y*_0_) is the initial position of the manipulated microsphere. The design of the actuator reduces the scope at each PID adjustment, thereby avoiding the escape of the manipulated microsphere.

**Figure 5:**
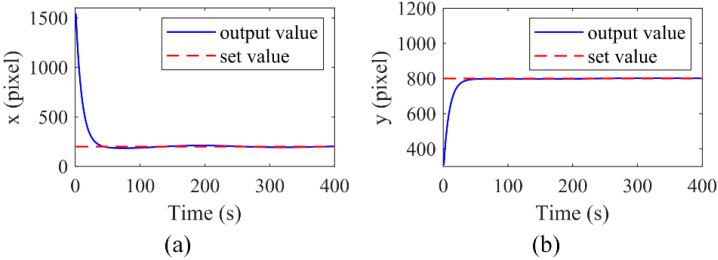
Numerical simulation results of the control system at the x-axis (**a**) and the y-axis (**b**), respectively.

In order to verify the efficiency and select the control parameters, a numerical simulation was performed. Here, only a PI control was used, and the PI parameters of both the x-axis and y-axis directions were set to be consistent. The PI parameters were selected as: proportional coefficient *K_p_* = 0.01 and integral coefficient *K_i_* = 0.1. The targeted location of the manipulated microsphere is (200, 800), and the initial position is (1500, 300). The curves of the model output at the x-axis and y-axis direction are shown in Figure 5a,b, respectively. The results show that the manipulated microsphere finally stays at the target position within 50 s and indicate that the microsphere can reach the set position accurately under the PI control parameters.

## 4. Results and Discussion

### 4.1. Experimental Verification

Next, the PID controller and the designed actuator were integrated into the ODEP system. Combined with the image recognition approach, which can acquire the real-time location of the manipulated microsphere, proposed in our previous work [19], the precise automatic manipulation on the microsphere was performed as follows.

In this experiment, the inner radius and outer radius of the optical pattern were set as 20 μm and 25 μm, respectively. The PI parameters were set as: proportional coefficient *K_p_* = 0.01 and integral coefficient *K_i_* = 0.1. The coordinates of the initial position of the manipulated microsphere were (235, 468), and the coordinates of the target location were (635, 868). The amplitude and frequency of the AC voltage were set as 15 V and 300 kHz, respectively.

**Figure 6:**
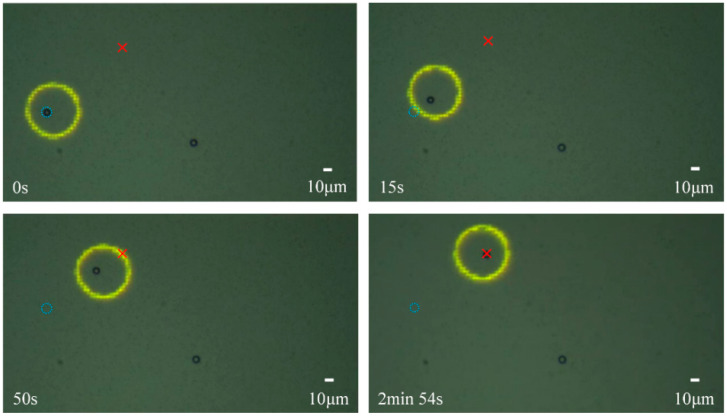
Moving process of the microsphere manipulation based on the designed control system. The yellow circle is the optical pattern, the red “×” is the target point and the blue dotted circle is the initial position.

The manipulation process and results are shown in Figure 6. The target location is marked with a red cross, and the initial position is indicated as a blue dotted circle. The manipulation process (Figure 6) demonstrates that the microsphere can accurately reach the target position.

**Figure 7:**
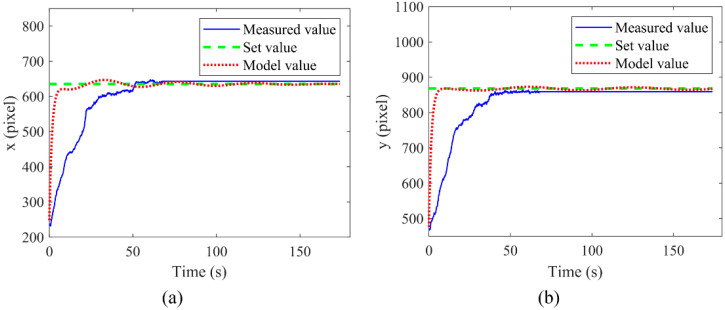
Diagrams of the model output and the actual output at the x-axis (**a**) and the y-axis (**b**), respectively.

Subsequently, the real-time position changes of the manipulated microsphere between the calculated results from the model and the actual, measured results were compared under the same PI parameters and set points, as shown in Figure 7. Figure 7a,b represent the time-dependent curves of the theoretical and the experimental coordinate values of the x-axis and y-axis during the operation, respectively. The results show that although the manipulation in the experiment has a hysteresis relative to the theoretical calculation, the manipulated microsphere was moved to the set position in a relatively short time both theoretically and experimentally. It is also validated that the model described above can accurately demonstrate the manipulation process of ODEP.

### 4.2. Accurate Manipulation

**Figure 8:**
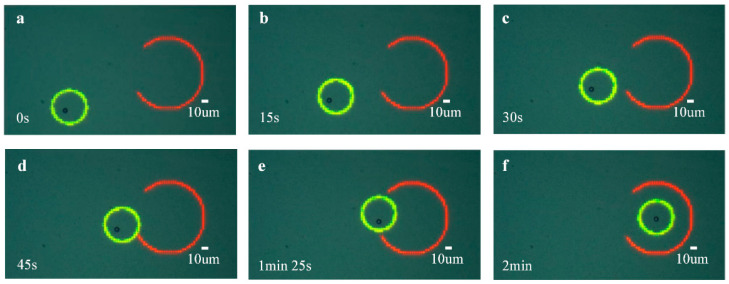
Diagrams of the manipulation process. The center of the red arc is the target position and the notch of the arc is the entrance of the optical pattern; The green circle is an annular optical pattern. (**a**–**f**) are the real-time status of automatic manipulation in chronological order.

In this section, the control method was used in different scenarios of precise manipulation. Achieving precise micromanipulation in narrow areas is a typical application of ODEP. Therefore, the first scenario is to move the manipulated microsphere through a narrow passage to the target position, as shown in Figure 8. The center of the red arc is the target location, whose coordinate values are (1300, 500), and the notch of the arc is the entrance of the manipulation of the annular optical pattern. Here, the red arc represents a barrier that prohibit passage. The radius and line width of the red arc are 40 μm and 3 μm, respectively. The inner radius and outer radius of the annulus optical pattern were set as 20 μm and 25 μm, respectively, and the amplitude and frequency of the AC voltage were set as 15V and 300 kHz, respectively.

Figure 8a–f show the manipulation processes in this scenario. In this experiment, a transition point was set at the center of the entrance of the red arc to ensure that the light pattern would not touch the arc wall during the manipulation. The results illustrate that the control method can accurately move the microsphere to the target position in this scenario with a position error approximating 0.

**Figure 9:**
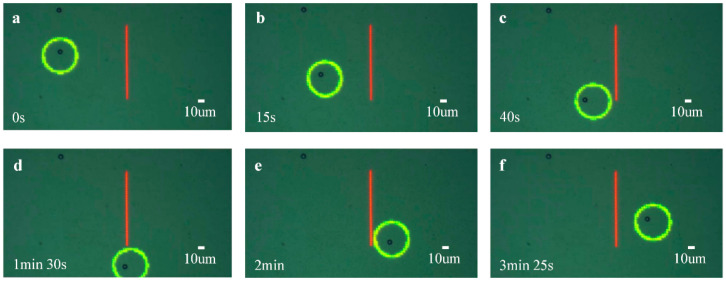
Diagrams of the manipulation process for obstacle avoidance. The red linear grating denotes the obstacle, and the green circle is the annular optical pattern. (**a**–**f**) are the real-time status of automatic manipulation in chronological order.

Obstacle avoidance is also a common scenario in ODEP micromanipulation, especially during the precise screening and isolation of living cells. Then, in another scenario, the control method to the micromanipulation was used in the application of obstacle avoidance. As shown in Figure 9, the red linear spot represents an obstacle, and the green circle is the annular optical pattern. Tthe inner radius and the outer radius of the annular optical pattern were set as 20 μm and 25 μm, respectively; the length and width of the linear spot were 90 μm and 2.5 μm, respectively. The initial position is to the left of the linear spot, and the target location is to the right of the linear spot. The amplitude and frequency of the AC voltage were set as 15 V and 300 kHz, respectively.

In this application scenario, the microsphere bypassed the red obstacle from the initial position to the target location. Similarly, to prevent the annular optical pattern from touching the linear spot during the manipulation, a transition potion was set below the linear spot, and the microsphere was first moved to this transition point and then to the target location. Figure 9a–f show the operation process in this scenario. The results demonstrate that the control method proposed in this paper can precisely move the microsphere to the target position across the obstacle, and the position accuracy obtained by the data analysis of the moving trajectory approximates 0.

## 5. Conclusions

In summary, this paper proposed an accuracy control method for ODEP manipulation by establishing the corresponding data-driven kinematic model. The displacements information of the optical pattern and the manipulated microsphere were obtained, respectively, through the data acquisition and image recognition methods under the designed optical patterns. Then, the kinematic model of the ODEP manipulation was established with the input of the optical pattern displacements and the output of the microsphere displacements. The kinematic model does not pay attention on the various forces applied on the manipulated object, avoiding the challenges of precise operation due to the unmeasurable nature of the ODEP force. Then, the control system of the ODEP manipulation was designed based on the kinematic model, and the control parameters were obtained through numerical simulations. The feasibility of the designed control system was verified by precise manipulation on microspheres, and the applications on two typical scenarios of the control system further demonstrates the feasibility of the control method in ODEP manipulation. This study provides a new model to accurately demonstrate ODEP manipulation and establishes a precise control method for ODEP manipulation, which is of great significance to further expand the application of ODEP. It also provides a general approach for the improvement of the manipulation accuracy of other optoelectronic tweezers. In the future, kinematic models based on a variety of optical patterns and operation modes will be established, making ODEP suitable for any occasion to achieve precise micromanipulation.

## Data Availability

Data underlying the results presented in this paper can be obtained from the authors upon reasonable request.
